# Acetylation of Lysine 382 and Phosphorylation of Serine 392 in p53 Modulate the Interaction between p53 and MDC1 *In Vitro*

**DOI:** 10.1371/journal.pone.0078472

**Published:** 2013-10-23

**Authors:** Or David Shahar, Ronen Gabizon, Oren Feine, Raphael Alhadeff, Assaf Ganoth, Liron Argaman, Elee Shimshoni, Assaf Friedler, Michal Goldberg

**Affiliations:** 1 The Department of Genetics, Alexander Silberman Institute of Life Sciences, the Hebrew University of Jerusalem, Jerusalem, Israel; 2 The Department of Organic Chemistry, the Hebrew University of Jerusalem, Jerusalem, Israel; 3 Department of Biological Chemistry, Alexander Silberman Institute of Life Sciences, Hebrew University of Jerusalem, Jerusalem, Israel; 4 The Interdisciplinary Center (IDC), Herzliya, Israel and Department of Biology and Environment, Faculty of Natural Sciences, University of Haifa-Oranim, Tivon, Israel; Dana-Farber/Harvard Cancer Institute, United States of America

## Abstract

Occurrence of DNA damage in a cell activates the DNA damage response, a survival mechanism that ensures genomics stability. Two key members of the DNA damage response are the tumor suppressor p53, which is the most frequently mutated gene in cancers, and MDC1, which is a central adaptor that recruits many proteins to sites of DNA damage. Here we characterize the *in vitro* interaction between p53 and MDC1 and demonstrate that p53 and MDC1 directly interact. The p53-MDC1 interaction is mediated by the tandem BRCT domain of MDC1 and the C-terminal domain of p53. We further show that both acetylation of lysine 382 and phosphorylation of serine 392 in p53 enhance the interaction between p53 and MDC1. Additionally, we demonstrate that the p53-MDC1 interaction is augmented upon the induction of DNA damage in human cells. Our data suggests a new role for acetylation of lysine 382 and phosphorylation of serine 392 in p53 in the cellular stress response and offers the first evidence for an interaction involving MDC1 that is modulated by acetylation.

## Introduction

 Genomic instability is a hallmark of cancer cells [1]. The primary cause of genomic instability is DNA damage [2]. Occurrence of DNA damage in a cell activates the DNA damage response (DDR), which is crucial for protecting the cells from genomic crisis. Proper activation of the DDR utilizes a complex, rapid and tightly regulated cascade of protein-protein interactions leading to cell-cycle arrest, DNA repair, apoptosis or cellular senescence [3-7]. 

The cellular response to DNA damage is driven by numerous post-translational modifications (PTMs) of histones and other proteins, which include phosphorylation, poly(ADP-ribosyl)ation, acetylation, ubiquitylation and sumoylation [7]. When the histones are post-transcriptionally modified, they can directly regulate the DDR by changing the chromatin structure at sites of DNA damage [8,9]. PTMs can also regulate the chromatin structure at sites of damage by modulating protein-protein interactions that are essential for the recruitment of different chromatin modifiers [7,10]. Indeed, protein-protein interactions, which are required for proper DNA repair, checkpoint activation and apoptosis are tightly regulated by PTMs [7]. 

**Figure 1:**
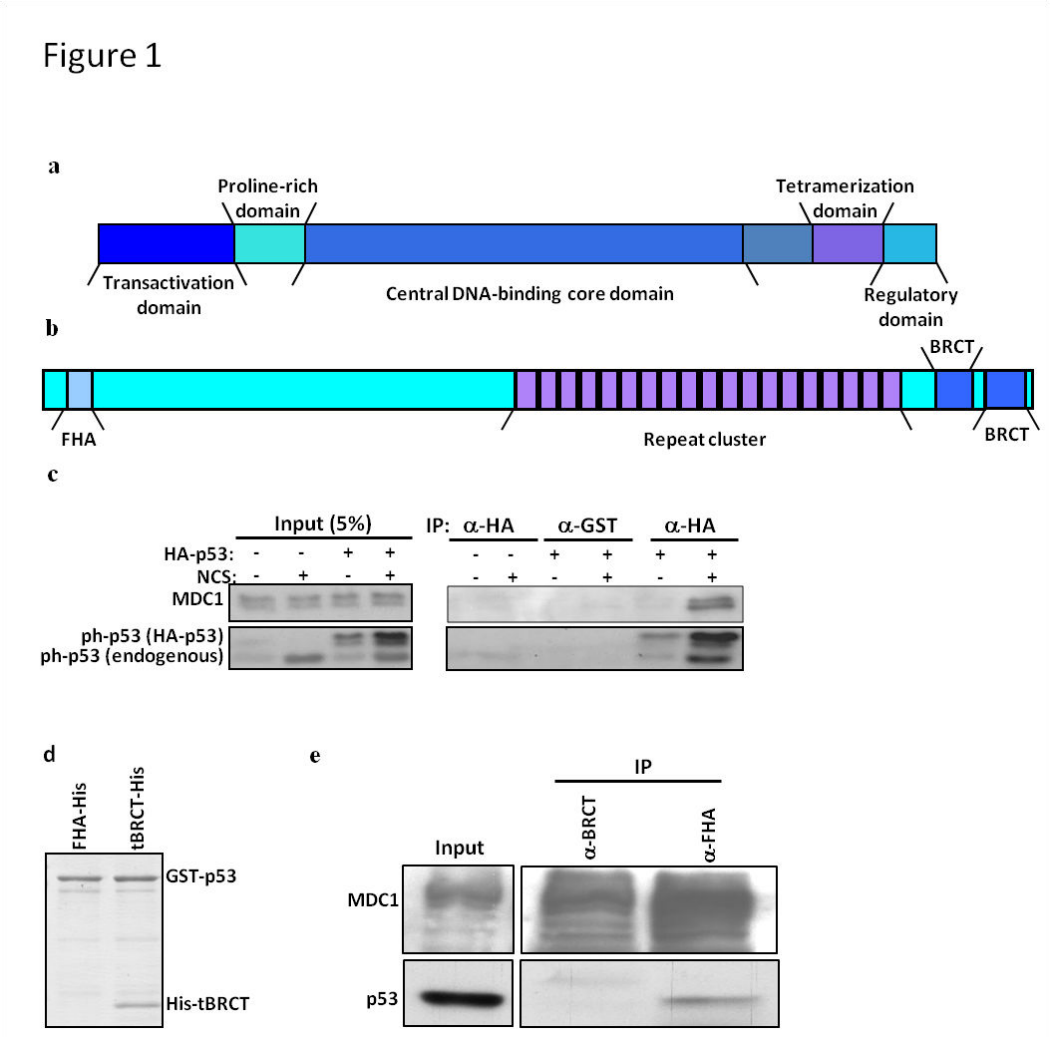
MDC1 and p53 interact following DNA damage through the tBRCT domain of MDC1. (a) A scheme of the different domains of p53. (b) A scheme of the different domains of MDC1. Note that in A, and B the images are not to scale as p53 is about 5 times smaller than MDC1. (c) 293T control cells transfected with empty vector or cells overexpressing HA-tagged p53 were either untreated (-) or treated with NCS (+). Following 1 hr incubation, the proteins were extracted and subjected to co-IP using anti-HA antibodies. Control for the co-IP was done with anti-GST antibodies. Detection was done using antibodies directed against endogenous MDC1 and phopsho-Ser15 of p53 (ph-p53). (d) The tBRCT domain of MDC1 directly interacts with p53. GST pull-down assay was performed with GST-p53 and His-tBRCT or His-FHA. All recombinant proteins were expressed in bacteria. Proteins were separated on a SDS gel and stained with Coomassie blue. (e) Endogenous p53 and MDC1 interact. Protein extracts prepared from 293 cells that were induced with 5 Gray of ionizing radiation and left for recovery for 1 hr were used in an IP experiment using antibodies directed against MDC1-tBRCT (α-BRCT) or against MDC1-FHA (α-FHA). Bound proteins were detected in Western blot using anti-p53 antibodies. Inputs present 5% of the extract used in the experiment.

 The tumor suppressor protein p53 is the most frequently mutated gene in human cancers. p53 primarily functions as a transcription factor that induces growth arrest, repair, apoptosis or cellular senescence [11-15]. p53 is composed of multiple domains (Figure 1a): The N-terminal transactivation domain (N’) is mainly a binding platform for transcriptional coactivators [16]. It is followed by a proline-rich region, which is important for its growth suppression activity [17]. The p53 core domain is the sequence-specific DNA binding domain [18]. It is followed by a linker region with an embedded nuclear localization signal, a tetramerization (Tet) domain [19] and a C-terminal domain (CTD), which is a disordered negative regulatory domain [20]. p53 is tightly regulated at multiple levels. It is constantly degraded under normal growing conditions and its protein level is induced following cellular stress [21]. Moreover, a tetramer formation by p53 is crucial for its activity [19]. The activity of p53 is heavily modulated by a variety of PTMs, mainly acetylation and phosphorylation. Phosphorylation of p53 occurs mainly on serine and threonine residues located at the N` domain and the C-terminus of the protein [22-24]. Most of these phosphorylation events occur rapidly following cellular stress, although a few sites are constitutively phosphorylated in unstressed cells and dephosphorylated following DNA damage induction [23]. Phosphorylation of serine 392 (S392) in p53 is induced upon DNA damage and has a role in the activation of the sequence specific DNA binding of p53 [25-29] and probably also in the stabilization of the p53 tetramer [30,31], and thus it is essential for proper activity of p53 [19]. Six lysine residues in the CTD of p53, including lysine 382 (K382) are acetylated, resulting in the activation of sequence-specific binding of p53 to DNA, transcriptional activation and stabilization of p53 [23,32,33].

 MDC1 is a central player in the DDR. It recruits different proteins to sites of DNA damage and thus facilitates their activation upon DNA damage induction. MDC1 regulates the G2/M and intra-S phase DDR checkpoints [34-37] and it plays a role in DNA double-strand break (DSB) repair [38-41]. DSBs are the most severe form of DNA damage, since if not responded to properly they may result in genomic instability [42]. MDC1 is a large protein composed of several protein-protein interacting modules (Figure 1b; [43]). These include a forkhead associated (FHA) domain, which is a phospho-protein binding module commonly found in signaling proteins [44] and tandem BRCA1 C-terminal (tBRCT) repeats. BRCT domains are protein-protein interaction modules that are found in proteins involved in the DDR. tBRCT domains may act as phospho-protein binding modules [45-47]. 

 p53 and MDC1 have overlapping roles in the DDR. Both proteins play a role in DSB repair. MDC1 was demonstrated to be involved in both NHEJ [38,39] and in HR [40,41], and p53 suppresses HR [48-52]. In addition, p53 and MDC1 have a role in checkpoint activation and in apoptosis [53-55]. Moreover, MDC1 and p53 are part of the same protein complex [54] and they directly bind similar DDR members, such as Rad51 [40,48] and 53BP1 [56,57]. Furthermore, downregulation of MDC1 results in decreased and delayed p53 stabilization and phosphorylation in response to DNA damage [58].

 Since p53 and MDC1 have overlapping roles in the DDR, we analyzed, *in vitro*, whether these proteins interact and how the interaction is molecularly regulated. We present evidence that human p53 and MDC1 directly interact through the tBRCT domain of MDC1 and the CTD of p53. We further show that both acetylation of K382 and phosphorylation of S392 in p53 enhance the interaction between p53 and MDC1. Finally, we reveal that the p53-MDC1 interaction is augmented upon the induction of DNA damage in human cells. Our results suggest a new role for acetylated K382 (Ac-K382) and phosphorylated S392 (pS392) of p53 in the cellular stress response and provide, if they also occur *in vivo*, the first evidence for an interaction involving MDC1 that is modulated by acetylation.

## Results

###  The p53-MDC1 interaction in cells is augmented upon DNA damage induction

Previous work has shown, using co-IP reactions, that endogenous p53 and MDC1 are part of the same complex in human cells [54]. Since the activity of p53 is largely dependent on genotoxic stress and p53 is constantly degraded in the absence of stress [21], we analyzed whether the p53-MDC1 interaction in human cells is controlled by genotoxic stress. To overcome the low protein levels of p53 and to study the effect of DNA damage on the interaction we have cloned p53 fused to a HA-tag (HA-p53), and overexpressed it in 293T cells. We then performed co-IP experiments using anti-HA antibodies to retrieve HA-p53. Following neocarzinostatin (NCS) treatment, which induces DSBs, both endogenous p53 and overexpressed HA-p53 were phosphorylated, as demonstrated by the stress-related phosphorylation on serine 15 of p53 (ph-p53 antibodies; Figure 1c), implying that the DDR was activated. MDC1 was retrieved by the anti-HA antibodies only following NCS treatment as detected when blotting with a specific antibodies directed against MDC1 (Figure 1c). Hence, DNA damage induction enhances the binding between p53 and MDC1.

###  The tBRCT domain of MDC1 binds p53

MDC1 is an adaptor protein that binds many proteins during the DDR through several protein-protein interacting modules. Most interactions involving MDC1 occur via the FHA and the tBRCT domains of the protein [43]. In order to map the p53 interacting domain in MDC1, we performed a glutathione S-transferase (GST) pull-down assay to analyze the binding between bacterially expressed full-length p53 fused to GST (p53-GST) and the tBRCT or the FHA domains of MDC1 (MDC1-tBRCT and MDC1-FHA, respectively) fused to a His-tag (His-tBRCT and His-FHA, respectively). Purification of bacterially expressed His-tBRCT resulted in a mixture of soluble aggregates and monomeric His-tBRCT (Figure S1). We thus separated these fractions by gel filtration (Figure S1) and used the monomeric His-tBRCT in further experiments. p53-GST pulled down His-tBRCT (Figure 1d) but failed to pull down His-FHA (Figure 1d). Taken together, these results demonstrate that the p53 binding region of MDC1 is MDC1-tBRCT. A support for tBRCT-MDC1 being the p53 binding domain of MDC1 was obtained when we analyzed the interaction between endogenous p53 and MDC1 upon DNA damage induction by performing co-IP experiments using two different anti-MDC1 antibodies to co-IP p53 from protein extract. Notably, we found that antibodies directed against MDC1-FHA co-IP p53 much stronger than antibodies directed against MDC1-tBRCT, which hardly co-IP p53 (Figure 1e). This difference may result from a competition between the anti-MDC1-tBRCT antibodies and p53 for the binding to MDC1. Therefore, both results suggest that MDC1-tBRCT is the p53 binding region of MDC1

###  The CTD of p53 binds MDC1

**Figure 2:**
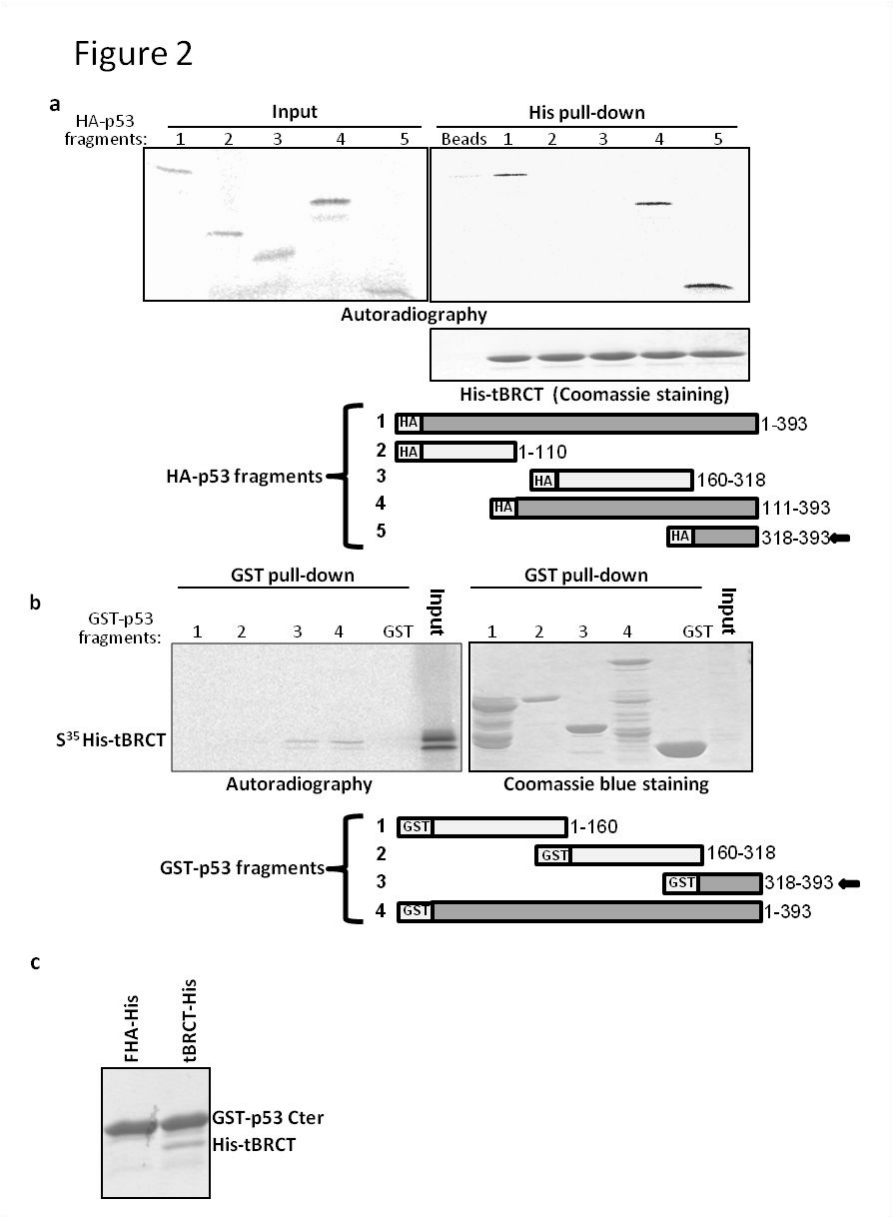
A C-terminus region (a.a. 318-393) of p53 directly binds MDC1-tBRCT. (a) His-tBRCT retrieves p53 fragments consisting a.a. 318-393: Bacterially expressed His-tBRCT was incubated with different radio-labeled fragments of p53-HA expressed in reticulocytes (for details see schematic representations below). Following His pull-down reactions the labeled p53 fragments (in the input or those retrieved by His-tBRCT) were visualized by autoradiography. (b) p53 fragments containing a.a 318-393 bind tBRCT-MDC1: Fragments of p53 fused to GST (for details see schematic representations below) were expressed in bacteria and purified. Following incubation with radio-labeled His-tBRCT and GST pull-down reactions, His-tBRCT visualized by using autoradiography. Input is 5% of His-tBRCT added to the reaction. The same gels were used for autoradiography and Coomassie blue staining in B. (c) GST pull-down using a.a. 318-393 of p53 fused to GST (GST-p53Cter) for His-FHA or His-tBRCT, followed by Coomassie blue staining.

Next, we aimed to map the region of p53 that binds tBRCT-MDC1. His pull-down experiment was done with bacterially expressed His-tBRCT and radio-labeled fragments of p53 fused to an HA-tag that were expressed in an *in vitro* reticulocyte system. Full length p53 or fragments containing the C-terminus of p53 (consisting the Tet domain and the CTD; residues 318-393) bind MDC1-tBRCT (Figure 2a), indicating that the C-terminus of p53 mediates the binding with MDC1. We have also expressed different fragments of p53 fused to GST in bacteria and conducted GST pull-down for radio-labeled His-tBRCT of MDC1 (appears on the gel as two adjacent bands; Figure 2b). In agreement with the His pull-down reaction shown in Figure 2a, His-tBRCT is only retrieved when pulled down with GST-p53 or with a fragment of p53 containing the protein C-terminus sequences (residues 318-393) fused to GST (Figure 2b). It is important to mention that the GST fusion, containing a.a. 1-110 of p53 is highly degraded, despite performing ion exchange and gel filtration (data not shown). We further verified that the interaction is mediated by the C-terminus of p53 by performing a GST pull-down with bacterially expressed GST fusion containing residues 318-393 of p53 and His-tBRCT (Figure 2c). This GST fusion pulled down His-tBRCT but failed to pull down His-FHA (Figure 2c), as was the case with full length p53 (Figure 1d). 

**Figure 3:**
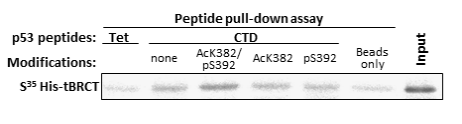
The CTD of p53 interacts with MDC1-tBRCT and this interaction is modulated by acetylation of K382 and phosphorylation of S392 in p53. Peptide pull-down assay was performed with peptides corresponding to the CTD of p53 (a.a 361-393) that were either without PTMs (none), acetylated on K382 (AcK382), phosphorylated on S392 (pS392) or containing both PTMs, or with a peptide corresponding to the Tet domain of p53 in the presence of radio-labeled His-tBRCT. Bound proteins were visualized using autoradiography.

 The C-terminus of p53 (residues 318-393), which we found to bind MDC1-tBRCT (Figure 2) is composed of two domains, namely, the Tet domain (residues 325-356) and the CTD (residues 361-393; [22,23]). In order to further map the interaction, we synthesized peptides corresponding to both the Tet domain and the CTD of p53 (Tet and CTD peptides, respectively). A peptide pull-down assay, performed with these peptides incubated with bacterially expressed His-tBRCT, indicated that the interaction occurs through the CTD and not via the Tet domain of p53 (Figure 3; Tet and CTD peptides; compare beads only to the Tet and CTD (none modification) peptides). 

###  The interaction between p53 and MDC1-tBRCT is direct

The interaction between p53 and MDC1-tBRCT is direct since we demonstrated that they interact by using purified proteins that were expressed either in bacteria or in the *in vitro* reticulocyte system or by using synthesized peptides in the peptide pull-down assay (Figures 1d, 2 and 3). 

###  Acetylation of lysine 382 and phosphorylation of serine 392 in p53 modulate the interaction with MDC1

We revealed a direct interaction between bacterially over-expressed MDC1-tBRCT and the CTD of p53 (Figures 1d and 2). PTMs that play an important role in the DDR [7], are, in many cases, absent in proteins that are expressed in the bacteria. However, PTMs may still modulate this interaction. The CTD of p53, which we found to interact with MDC1 (Figures 2 and 3) undergoes such modifications. Specifically, K382 is acetylated and S392 is phosphorylated following DNA damage [59,60]. In order to test if these PTMs modulate the interaction between p53 and MDC1, we synthesized p53 CTD peptides containing Ac-K382 and pS392 or peptides containing either Ac-K382 or pS392. Peptides are ideal for studying the effect of PTMs on protein-protein interactions since they allow introducing a specific modification in a desired residue. In order to examine the ability of MDC1-tBRCT to pull-down unmodified p53 CTD peptide and a peptide containing both Ac-K382 and pS392 we performed a GST pull-down assay with MDC1-tBRCT fused to GST (GST-tBRCT). Only the p53 CTD peptide that contained pS392 and Ac-K382 was retrieved by GST-tBRCT (Figure S2a). The inability to detect binding between the unmodified p53 CTD peptide and GST-tBRCT (Figure S2a) may be since this interaction is weak and requires a higher peptide concentration, as should be obtained when peptide pull-down assays were performed. Therefore, we have implemented a peptide-pull down to analyze the binding between endogenous MDC1 and the unmodified p53 CTD peptide or the peptide containing both Ac-K382 and pS392. Both peptides retrieved MDC1 from a nuclear HeLa extract, while the Tet peptide failed to bind MDC1 (Figure S2b). However, although the modified peptide seems to bind stronger MDC1 compared to the unmodified peptide (Figure S2b), this method is not sensitive enough for full and quantitative addressing this point. Notably, the Ted peptide, which does not bind MDC1, did retrieve endogenous p53 from the extract, indicating that the peptide is active and can interact with p53 (Figure S2b). Next, we have carried out a peptide pull-down assay using the different p53 CTD peptides and MDC1-tBRCT. This pull-down assay revealed that the peptide containing both Ac-K382 and pS392 binds MDC1-tBRCT stronger than the unmodified or the single modified peptides (Figure 3). However, we could not detect the contribution of each PTM on the binding using this method (Figure 3). Taken together, the results above suggest that these PTMs modulate *in vitro* the interaction between the CTD of p53 and MDC1-tBRCT.

**Figure 4:**
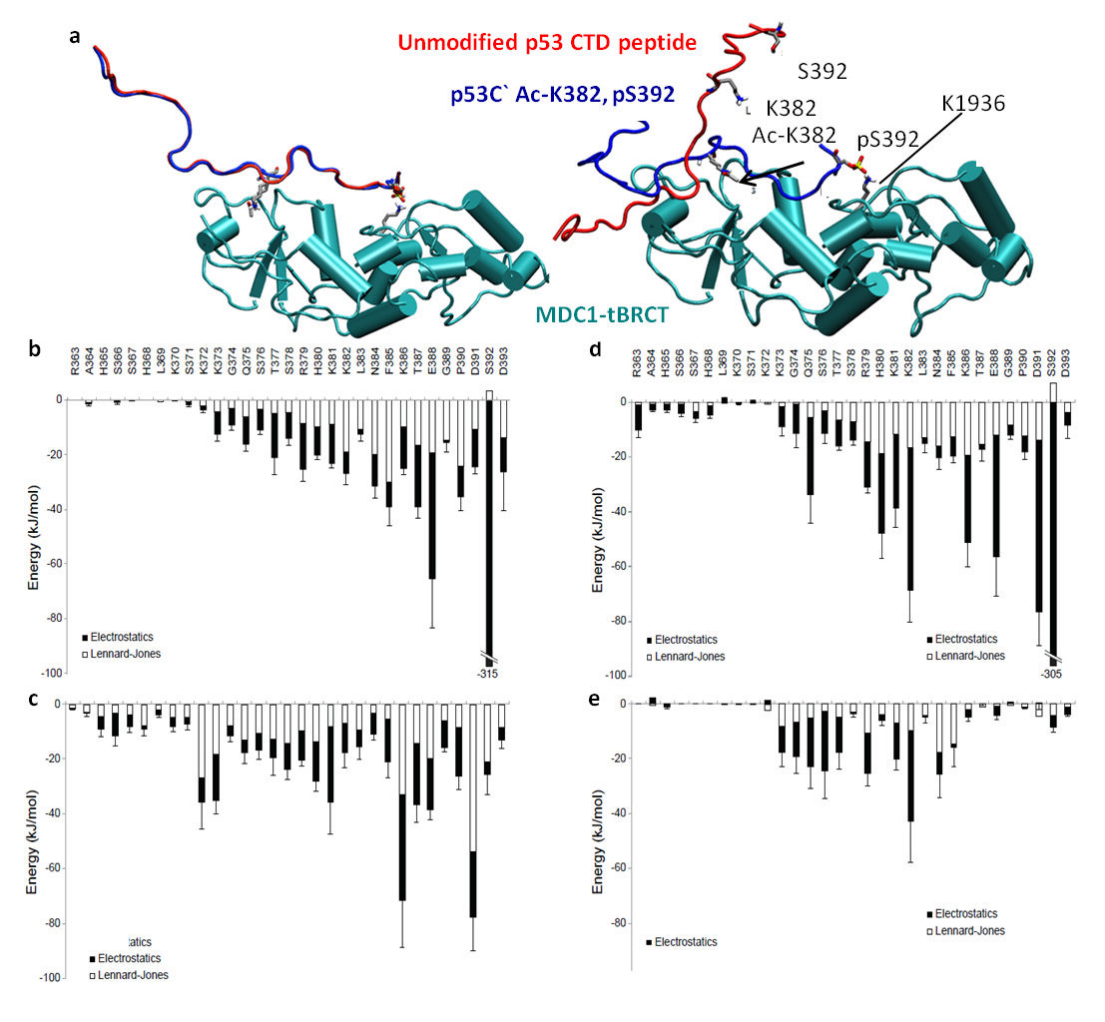
In p53 CTD, acetylation of K382, phosphorylation of S392 or both, contribute to the binding to MDC1-tBRCT. (a) Cartoon representations of the p53 CTD peptides (Acetylated and phosphorylated peptide in blue or unmodified peptide in red), MDC1-tBRCT in cyan and phosphorous atom in gold. Left - initial conformations; right - representative snapshots of the molecular dynamics simulations. (b-e) Potential energy of the interactions between p53 CTD peptides and MDC1-tBRCT; The Lennard-Jones and the electrostatic contributions of each residue are shown in white and black, respectively. The peptides: (b) Ac-K382 and pS392. (c) Ac-K382. (d) pS392. (e) Unmodified. Error bars represent the standard deviation of the mean for the sum of the interactions.

 Since peptide pull-down is not a quantitative assay, the partial contribution of each modification itself could not be determined. To do so, we conducted molecular dynamics (MD) simulations where we simulated the binding of MDC1-tBRCT to an unmodified CTD peptide, a CTD peptide with Ac-K382 and pS392 or with peptides having one of these PTMs. First, we assessed the stability of the tBRCT protein during the simulations by analyzing its backbone root mean square deviation, and found that it was stable, exhibiting low values (~ 0.25nm) with no major fluctuations (data not shown). We have simulated MDC1-tBRCT at the presence of p53 CTD peptide with Ac-K382 and pS392 and followed the sterical conformations of the peptides. At the initial conditions of the simulations, the peptides, regardless of the PTM state, were in close proximity to MDC1-tBRCT (Figures 4a left, b, Figure S3, Videos S1-S5). Interestingly, whereas the double-modified peptide remains attached to MDC1-tBRCT (Figure 4a right, Videos S1 and S2), the unmodified peptide detaches from MDC1-tBRCT (Figure 4a right, Video S3). These results reinforce our peptide pull-down assay data (Figure 3), demonstrating that Ac-K382 and pS392 modulate the interaction between the CTD of p53 and MDC1-tBRCT.

 The simulation of the binding between MDC1-tBRCT and the CTD peptide of p53 containing Ac-K382 and pS392 revealed that the binding is strengthened between residues 379-393 of p53 (Figure 4b, Figure S3). Notably, the largest negative potential energy thrives from electrostatics interaction between pS392 of p53 and MDC1-tBRCT, demonstrating that pS392 serves as an anchor for this interaction (Figure 4b). 

 Next, we studied the contribution of each modification to the interaction by performing simulations of MDC1-tBRCT in the presence of p53 CTD peptides containing Ac-K382 or pS392 or with an unmodified p53 CTD peptide. The energy calculations between MDC1-tBRCT and the p53 CTD peptides containing either Ac-K382 or pS392 showed that both peptides interact with MDC1-tBRCT via similar residues as the double modified p53 CTD peptide (residues 370-393; Figure 4b-d, Figure S3, Videos S4 and S5). However, intriguingly, as demonstrated in Figure 4a, the unmodified peptide is not tightly bound to MDC1-tBRCT and it exhibits a completely different energy pattern. The unmodified peptide loosely binds to MDC1-tBRCT and the interaction is mediated by other residues compared to the modified peptides, mainly residues 373-385 in p53 (Figure 4e). Interestingly, according to the computational analysis Lysine 1936 (K1936; Figure 4a) in MDC1 is in close proximity to the pS392 in p53 and takes part in the phospho-mediated interaction. This result is in line with the known importance of K1936 for the ability of MDC1-tBRCT to mediate phospho-dependent interactions [61]. Taken together, our data imply that acetylation of K382 and phosphorylation of S392 enhance the *in vitro* binding of p53 to MDC1.

###  The secondary structure of the p53 CTD peptides does not change due to acetylation of K382 and phosphorylation of S392

**Figure 5:**
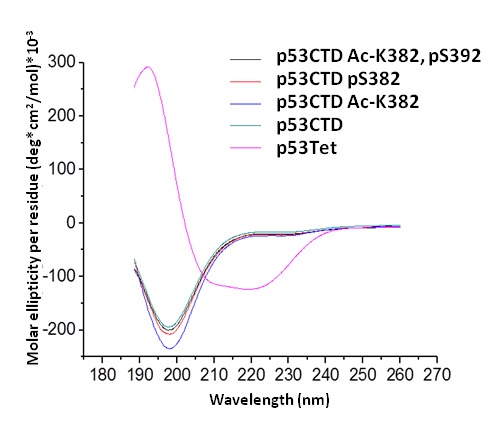
Secondary structure of the CTD of p53. CD spectra of p53 Tet peptide and p53 CTD peptides that are either unmodified, containing both AcK382 and pS392 or containing either AcK382 or pS392.

The p53 CTD is intrinsically disordered and contains no permanent secondary structure while the Tet domain of p53 has a spectrum of a folded peptide, with mostly alpha helical content ([62,63] and Figure 5). Phosphorylation introduces negative charge on proteins while acetylation removes a positive charge from proteins. Hence, acetylation of K382 and phosphorylation of S392 in p53 may enhance the interaction with MDC1-tBRCT due to a change in the peptide structure. We used circular dichroism (CD) to uncover the secondary structure of the modified CTD peptides and found that whereas the Tet domain has α-helical structure as published [62]. All the post-transcriptionally modified peptides have a typical spectrum of unfolded proteins (Figure 5). This suggests that Ac-K382 and pS392 of p53 modulate the interaction with MDC1-tBRCT by changing the electrostatic charge of the CTD of p53 and not by providing a more defined structure to support the interaction. 

## Discussion

 Our data indicate that the CTD of p53 and MDC1-tBRCT directly interact and that acetylation of K382 and phosphorylation of S392 in p53, which occur upon genotoxic stress enhance the *in vitro* binding of p53 to MDC1. 

**Figure 6:**
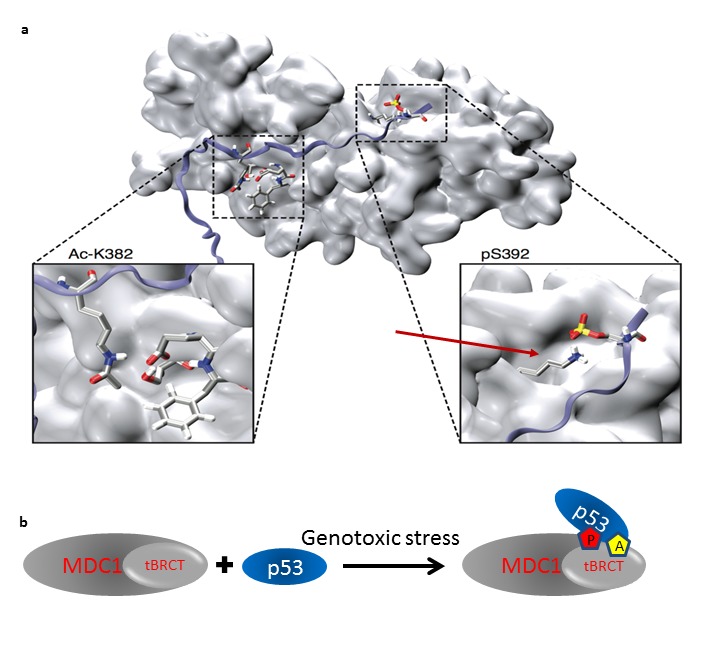
Suggested model and visualization of the p53-MDC1 interaction. (a) MD derived interactions. tBRCT is shown in surface representation in light gray and the p53 CTD peptide is shown as a blue ribbon. Zoom-in panels of the Ac-K382 and pS392 are shown below; note that in the zoom-in panels the viewer angle is slightly rotated for visualization convenience. Red arrow points K1936 in MDC1. (b) Following genotoxic stress p53 (blue) undergoes K382 acetylation (red pentagon) and S392 phosphorylation (yellow pentagon). These residues mediate the interaction with MDC1 (gray) through its tBRCT domain (light gray).

 Biochemical studies aimed to characterize protein-protein interactions allow mapping of the residues that are important for the interaction, revealing the conditions and the PTMs that modulate the interactions. Such findings may shed important light on the interaction and its regulation. However, if there is no resolved structure of the interaction, they do not provide information at the molecular level nor do they allow visualization of the interaction at atomic resolution. Studies based on computational analysis and dynamics provide atomically detailed understanding of protein-protein interactions. Yet, because computational methodologies are still relatively limited, combining them with other methods, synergistically, might prove to be highly beneficial. Here, we combined experimental studies with computational simulations to study the interaction between p53 and MDC1. We got consistent results from both approaches, strengthening the conclusions of the computational data. We could evaluate the relative importance for the interaction of each PTM by itself as well as analyze the additive value of the PTMs for the interaction. Moreover, the computational analysis suggests the involvement of K1936 (Figures 4, 6a) in MDC1 in the phospho-mediated interaction. This is consistent with the known importance of K1936 in tBRCT-MDC1 phospho-dependent interactions [61]. Following genotoxic stress, the CTD of p53 undergoes PTMs, and our results suggest a model by which acetylation of K382 and phosphorylation of S392 in p53 enhance the interaction of p53 with MDC1. This interaction of the CTD of p53 with MDC1 is direct and occurs through MDC1-tBRCT (Figures 1-4, 6).

 MDC1-tBRCT is involved in many protein-protein interactions [43,64]. This domain is a phospho-protein binding module [46,47,65] and therefore a subject of these interactions are regulated by phosphorylation [43,64]. Our results show that an addition PTM, acetylation, also modulates an interaction involving MDC1-tBRCT (Figures 3, 4, 6a and Figure S3). Acetylation of K382, as well of additional lysine residues in p53 upon genotoxic stress activates sequence-specific binding of p53 to DNA resulting in transcriptional activation and stabilization of p53 protein levels [20,23]. Our results imply that Ac-K382 has also a role in enhancing the binding of p53 to MDC1 (Figures 3, 4 and Figure S3). Since the p53-MDC1 interaction is augmented in the presence of these PTMs (Figures 3, 4 and 6), and MDC1 is rapidly recruited to sites of DNA damage [43,64], an additional role for these PTMs may be to enhance the binding of p53 to MDC1 and facilitate the recruitment of p53 to sites of damage, in order for it to be phosphorylated and thus activated by the kinases ATM and ATR [24]. 

 The CTD of p53 is modulated by many PTMs, including phosphorylation and acetylation [22-24]. The binding of the p53 CTD to DNA, RNA and other proteins is modulated by acetylation of this domain. Acetylation in p53 CTD promotes its binding to specific DNA sequences [33,38] and was found to either augment or impair protein-protein interactions involving the CTD of p53 (for example 66). Mdm2, the major regulator of ubiquitin-mediated degradation of p53, directly binds both the N` domain [21,67,68] and the CTD of p53 [66]. The binding to the N` domain inhibits the transcriptional activation function of p53 and allows ubiquitynation of p53 by MDM2 [21,67,68]. The interaction between the p53 CTD and Mdm2 is less efficient when p53 is acetylated [66]. This may be the mechanism by which PTMs on p53 CTD promote the dissociation of the Mdm2-p53 complex [69,70]. The bromodomain of the coactivator CBP (CREB binding protein), which is required for p53-induced transcriptional activation of the cyclin-dependent kinase inhibitor p21 in cell cycle arrest, binds specifically to the CTD of p53 at Ac-K382 [71]. Here we identify a novel interaction involving the CTD of p53 that is augmented by Ac-K382. We show that p53 CTD directly binds MDC1 and that this interaction is enhanced when K382 is acetylated (Figures 3, 4 and Figure S3). Future work is required in order to define if acetylation of K382 and phosphorylation of S392 in p53 are also involved in modulating the interaction between p53 and MDC1 in cells. 

 Previous work that studied the p53-MDC1 interaction found, consistently with this study, that tBRCT-MDC1 is the p53 binding region of MDC1. They claimed that the N` domain of p53 mediates the binding to MDC1 in human cells [54]. Additionally, Nakanishi et al., argued that the interaction between p53 and MDC1 does not occur in cells induced with DNA damage [54]. Our results clearly demonstrate that the p53-MDC1 interaction augments upon DNA damage induction. Not only that the PTMs found to modulate the interaction *in vitro* (Figures 3 and 4) are stress-related [59,60] but also we found that in human cells the interaction is stronger in cells induced with DNA damage (Figure 1). This is consistent with the very low protein levels of p53 in undamaged cells tightly regulated by the MDM2 feedback loop, and the elevation in p53 levels following DNA damage induction. Our extensive *in vitro* studies did not reveal a direct interaction between the N` domain of p53 and tBRCT-MDC1. This difference may be reconciled if the interaction involving the N` domain of p53 and tBRCT-MDC1 is modulated by PTM on either protein or due to the usage of HEK293T cells, which express T-antigen that affects p53, for the mapping of the interaction by Nakanishi et al., [54]. Moreover, the p53-MDC1 interaction may be multifaceted and involves several regions in both proteins and thus the tBRCT-MDC1 may interact with the CTD of p53, as we demonstrated, while the N` domain of p53 may also interact with MDC1. It cannot be ruled out that in the absence of DNA damage the low levels of p53 binds MDC1 via the N' domain of p53 to inhibit p53 pro-apoptotic activity as claimed by Nakanishi et al., [54]. Following DNA damage, the stabilized p53 undergoes K382 acetylation and S392 phosphorylation and binds MDC1 to facilitated proper DDR. 

## Materials and Methods

### Plasmids and Peptides

 GST-p53 plasmids (shown in Figure 2b) were a kind gift from T. Sheng [72]. To create the HA-p53 plasmids, the different p53 fragments (Figure 2a,d) were cloned into pcDNA3 containing HA tag. To create the His-tBRCT, the tBRCT domain of MDC1 was cloned to pHisParallel2 (Kindly given by Dr. Peter Sheffield, University of Virginia), and His-FHA was previously described [34]. Peptide synthesis was done on a Liberty peptide synthesizer with a Discover single mode microwave module from CEM (NC, USA), using standard Fmoc chemistry. Protected amino acids were purchased from Luxembourg Bio Technologies (Tel Aviv, Israel), Iris biotech GmbH (Marktredwitz, Germany), and Chem-Impex (Wood Dale, IL, USA). Coupling of modified residues to the resin was performed with Fmoc-N'-Acetyl-L-lysine and Fmoc-O-benzyl-L-phospho-serine (Novabiochem). Following coupling of Phospho-serine, all fmoc de-protections were carried out without microwave heating. For fluorescein labeling, the peptidyl resin was reacted with 5(6)-carboxyfluorescein (Molecular Probes^TM^) as described [73,74]. For biotin labeling, the peptidyl resin was reacted with biotin (Sigma, Israel) in 1:1 DMF: DMSO with Pybop activation. The peptides were purified on an ACE C8 semi-preparative column using gradients of 5% to 60% acetonitrile in water, with 0.1% trifluoroacetic acid (TFA) in both solvents. The identity of the peptides was validated using an Applied Biosystems Voyager-DE Pro MALDI TOF mass spectrometer and verified to be within ± 1 Da of the theoretical mass. The purity of all peptides was verified to be >95% for non-labeled peptides and >90% for fluorescein-labeled peptides by analytical HPLC. The purified peptides were lyophilized from 30% acetic acid to remove residual TFA. The concentrations of the peptides were measured by UV absorbance at 280 nm using extinction coefficients of 1490 M^-1^cm^-1^ for tyrosine and 5500 M^-1^cm^-1^ for tryptophan. Since the C-terminal peptides have no tyrosine or tryptophan in the sequence, a single tryptophan residue was added at their N-terminus. 

### Cell Culture, Extract Preparation, Protein Expression, and Purification

 293T and U2OS cells were cultured in Dulbecco's modified Eagle's medium supplemented with 10% fetal bovine serum, L-glutamine, penicillin, and streptomycin. High salt protein extracts were prepared according to a previous study [75].  

###  Antibodies

the commercial antibodies used in this study were: mouse monoclonal anti-HA (12CA5), mouse monoclonal anti p53 (DO-1), rabbit polyclonal anti phospho-serine 15 p53 (Cell Signaling). Anti-MDC1 antibodies included rabbit and sheep anti-MDC1 directed against the FHA and tBRCT domains of MDC1 [34] and mouse anti-MDC1, clone MDC1–50 (Sigma-Aldrich). Anti-GST antibodies (Sigma-Aldrich) were used as controls. 

### Protein expression and purification

His-tBRCT was expressed in BL21pS to Optical Density (OD) 0.3-0.4 (37°C, 200 RPM). 0.1% Glycerol and 0.1mM Potassium Glutamate were added to the medium. Following heat shock for 20-30 min at 42°C, the medium was transferred to 17°C, shaked at 200 RPM for 20 min and Isopropyl β-D-1-thiogalactopyranoside (IPTG) (0.8mM final concentration) was added. The bacteria were harvested after 8-10 hours in lysis buffer (50mM Tris pH=8, 5mM Imidazole, 10% glycerol, 0.1% triton X-100, DNase100mM + 0.1mg/ml Lysozyme, 1mM PI/AeBSF, 100mM NaCl, 5mM β-mercaptoethanol). Following microfluidizer the protein extract was purified on Superdex75 (GE healthcare), bound to Ni-NTA beads in: 50mM Tris pH=7.5, 25mM imidazole, 10% Glycerol, 0.1% Triton X-100, 100mM NaCl, and 5 mM bmercaptoethanol. His-tBRCT was eluted with 250mM imidazole. The eluted protein was loaded on a Superdex75 gel filtration pre-equilibrated with 25mM Tris pH=7.5, 50mM NaCl, 10% glycerol, 0.1% Triton X100. Fractions containing tBRCT-tBRCT were pooled. FHA-His was expressed in BL21pS (37°C, 200 RPM) to OD 0.6, and induced with 0.5mM IPTG. 

p53 fragments were expressed in Bl21pS and induced by adding 30mM IPTG. Expressed proteins were purified using Glutathione Sepharose 4B (GE Healthcare), followed by elution with 20mM Glutathione in PBS. Eluted proteins were dialyzed against dialysis buffer containing 10% Glycerol and 1mM Dithiothreitol (DTT) in PBS. The N-terminus of p53 was taken for further purification steps using Gel filtration on column Superose 12 (GE Healthcare) 97 x 1.6 cm (~200ml) Buffer: 20mM TricHCl pH=7.5, 0.1M NaCl, 10% Glycerol, 10mM β-mercaptoethanol. This was followed by Anion exchange on column (GE healthcare) resource 15Q 1ml column and elution with NaCl gradient. 

### In vitro translation reaction

Radioactively-labeled proteins were transcribed and translated for 90 min in a coupled in the TNT vitro reticulocyte system by Promega (WI, USA) at 30°C in the presence of S35 EasyTag Express Labeling Mix by Perkin-Elmer (MA,USA).

### Immunoprecipitation and GST pull-down assays

GST pull-down assays were done with 20 μg of the indicated bacterially expressed and purified GST fusion proteins and glutathione-Sepharose 4 Fast Flow beads (Amersham Biosciences). IPs were done with the indicated antibodies and protein A- or G-Sepharose beads (Santa Cruz Biotechnology or Roche Applied Science). High salt protein extracts (1–2 mg) were added to the IP or GST pull-down assays. Beads were washed extensively with wash buffer (20 mM HEPES, pH 7.4, 0.2 mM EDTA, 0.5 mM dithiothreitol, 0.2% Triton X-100, 150 mM NaCl) and bound proteins were subjected to SDS-PAGE and Western blots. 

### In vitro binding assay between recombinant proteins

 20 μg of recombinant GST-p53 aa 1-393 or GST-p53 a.a. 318-393 were incubated with glutathione-Sepharose 4 Fast Flow beads for 1 h at 4°C. After incubation for 2h at 4°C with His-FHA or HIS-tBRCT and intensive washes, the bound fraction was boiled for 10 min and subjected to SDS-PAGE and Coomassie blue staining.

### Histidine pull-down assay

 Ni–NTA magnetic beads (QIAGEN Inc. CA, USA) suspension (50ml) was added to 0.02 mg of His-tagged proteins diluted in 0.5 ml of His wash buffer (50mM NaH2PO4, 300mM NaCl, 20mM imidazole, and 0.05% Tween-20, pH=8.0), and the suspension was incubated on an end-over-end shaker for 30 min at 4°C. After removing the supernatant using a magnetic separator, 0.5 ml of the buffer was added. After mixing, the tubes were placed on magnetic separator for 1 min, and then the buffer was removed. 0.5 ml interaction buffer was added and incubated on an end-over-end shaker for 1 hour at RT, and again, the supernatant was removed on a magnetic separator and washed twice with 0.5ml of interaction buffer. Following washes, samples were boiled in sample buffer, run on gel, and subjected to autoradiography.

### Peptide pull-down assay

 200μl of 7.5μM biotin-labeled peptide solution was incubated with 10μl Avidin-conjugated magnetic beads, and 5μL of radioactively labeled tBRCT-His in the presence of 20μl of streptavidin-coated Dynabeads M-280 (Dynal). Beads were washed extensively with wash buffer (20mM HEPES, pH 7.4, 0.2mM EDTA, 0.5mM DTT, 0.2% Triton X-100, 150mM NaCl), and bound proteins were subjected to SDS-PAGE and exposed to radio autography for 3 days. 

### Systems set-up for molecular dynamics

 Four simulation systems were prepared, each contained a complex of a protein domain composed of two BRCT repeats (PDB entry 3K0H, chain A) and CTD-p53’s peptide (unmodified, when K382 is acetylated, when S392 is phosphorylated, and when both K382 is acetylated and S392 is phosphorylated). We used the data obtained from the solved structure between the BRCT of MDC1 and a peptide corresponding to the C-terminus of γH2AX [61] and replaced the C γH2AX peptide with the CTD-p53 peptide. The protein-peptide complex was embedded in a box containing the SPC water model [76], which extended to at least 1.2 nm between the protein-peptide structure and the edge of the box K^+^ and Cl^-^ ions were randomly added to each simulation box, to neutralize the system at a physiological salt concentration of 0.1 M. Then, each system was subjected to rigorous energy minimization using the steepest descent algorithm and tolerance of 1000 kJ.mol-^1^.nm^-1^, followed by a minimization using the conjugated gradient algorithm with a sequential decreasing convergence from 100 to 10 kJ.mol^-1^.nm^-1^. After the minimization phase, each system underwent an equilibration stage under positional restraints using a harmonic force constant. The equilibration procedure began with a force constant of k = 1000 kJ.mol^-1^.nm^2^ for 100 ps, then a force constant of k = 500 kJ.mol^-1^.nm^2^ for 100 ps, and another 100 ps of an unrestrained MD run. After the positional restraint equilibration, all systems were submitted for unbiased MD runs. 

### Molecular dynamics details

 All systems were subjected for at least 3 independent MD runs for proper statistics (for each system, one long run of > 30 ns and other shorter runs of > 10 ns were performed). All MD simulations were conducted using version 4.0.7 of the GROMACS package [77,78], employing an extended version of the GROMOS53a6 force field [79]. Parameters for the acetylated lysine were adopted from [80], whereas those for the phosphorylated serine were taken from the GROMOS43a1 force field and implemented to the GROMOS53a6 force field. The simulations were conducted using the LINCS algorithm [81] to constrain bond lengths and angles of hydrogen atoms, allowing a time step of 2 fs. Simulations were run using V-rescale temperature coupling at 310K employing a coupling constant of τ = 0.1 ps. Pressure was kept constant at 1 bar by applying an isotropic coupling with a coupling constant of τ = 2 ps using the Parrinello-Rahman pressure coupling [82]. A cutoff of 1.2 nm was used for van der Waals interactions; long range electrostatic interactions were computed using the PME algorithm [83]. Replica systems were generated the same starting configuration of each protein-peptide combination, but with different initial velocities applied.

### Circular Dichroism spectroscopy

Circular Dichroism (CD) spectra were recorded on a JASCO J-810 Spectrophotometer (JASCO, Japan) at 25°C. Peptides were diluted to 20-40μM in NaPi 25mM pH=7.5, 31mM Na_2_SO_4_, and their CD spectra were measured. Five spectra were measured and averaged for each peptide.

## Supporting Information

Figure S1 **His-tBRCT expression and purification.** (a) Coomassie Blue staining of His-tBRCT expressed in bacteria and purified using nickel beads. Shown are the elution fractions following incubation with imidazole. (b) His-tBRCT purification using gel filtration. The peak on the left reflects the His-tBRCT aggregates and the peak on the right is composed of the monomer. Monomer His-tBRCT was purified for biochemical assays.
(TIF)

Figure S2 **Interaction between MDC1 and p53-CTD peptides.** (a) GST pull-down with GST-tBRCT (described in Goldberg et al, Nature, 2003, 421:952-6) with the unmodified p53 CTD peptide, CTD peptide containing both Ac-K382 and pS392 or the Tet peptide. All peptides are fused to His-Tag. (b) Peptide pull-down assay was done using the unmodified p53 CTD peptide, CTD peptide containing both Ac-K382 and pS392 or the Tet peptide, to pull-down endogenous MDC1 and p53 from nuclear HeLa extract (Computer Cell Culture Centre). (A, B) Proteins retrieved in the pull-down assays were subjected to SDS-PAGE. Membranes were blotted with the indicated antibodies.
(TIF)

Figure S3 **Interaction between tBRCT-MDC1 and the p53-CTD peptides.** Representative snapshots from Molecular Dynamics simulations of the tBRCT-MDC1 and the p53-CTD peptides (a) Ac-K382 and pS392, (b) Ac-K382 or (c) pS392. (d) Unmodified CTD peptide.tBRCT-MDC1 is shown in surface representation and p53-CTD peptide is shown as a blue ribbon.
(TIF)

Movie S1 **Overlap of the double modified peptide (blue) and the unmodified peptide (red).** Note that whereas both peptides had the same initial position in space, the unmodified peptide detaches with time while the modified peptide is bound to MDC1-tBRCT and that the acetylated K382 and especially phosphorylated S392 are important for the interaction.
(MPG)

Movie S2 **The double modified peptide.**
(MPG)

Movie S3 **The unmodified peptide.**
(MPG)

Movie S4 **The K382 acetylated peptide.**
(MPG)

Movie S5 **The S392 phosphorylated peptide.**
(MPG)
