# Supplementary figures and images for: Acetylation of Lysine 382 and Phosphorylation of Serine 392 in p53 Modulate the Interaction between p53 and MDC1 In Vitro
Source: PLoS One. 2013 Oct 23;8(10):e78472. doi: 10.1371/journal.pone.0078472 (PMC3806821; doi:10.1371/journal.pone.0078472)

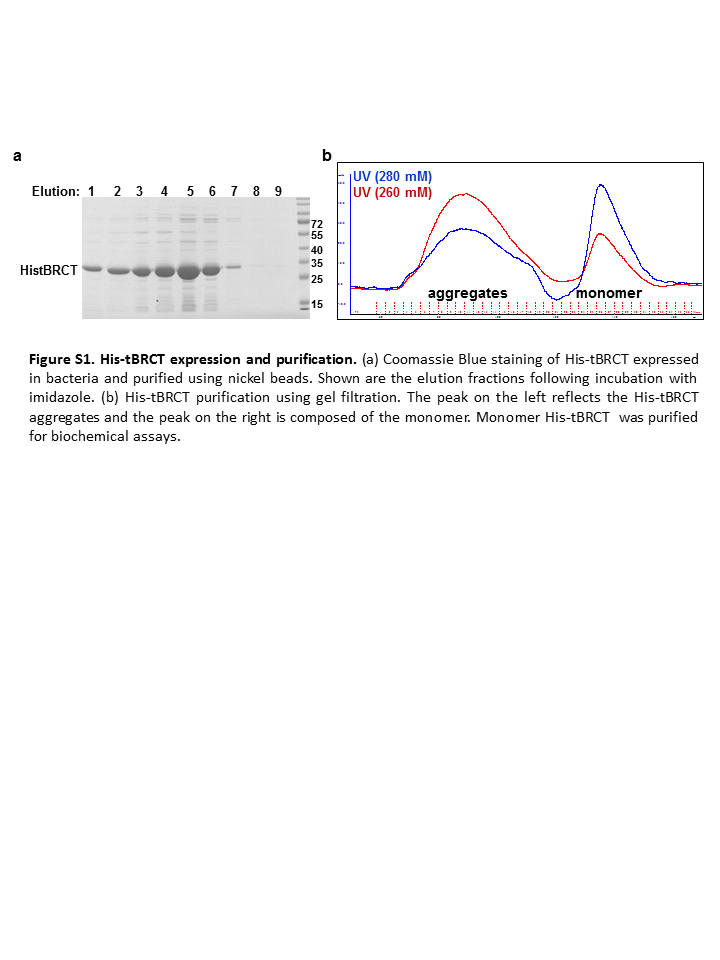

Supplement: Figure S1 — His-tBRCT expression and purification. (a) Coomassie Blue staining of His-tBRCT expressed in bacteria and purified using nickel beads. Shown are the elution fractions following incubation with imidazole. (b) His-tBRCT purification using gel filtration. The peak on the left reflects the His-tBRCT aggregates and the peak on the right is composed of the monomer. Monomer His-tBRCT was purified for biochemical assays. (TIF) [file pone.0078472.s001.tif]

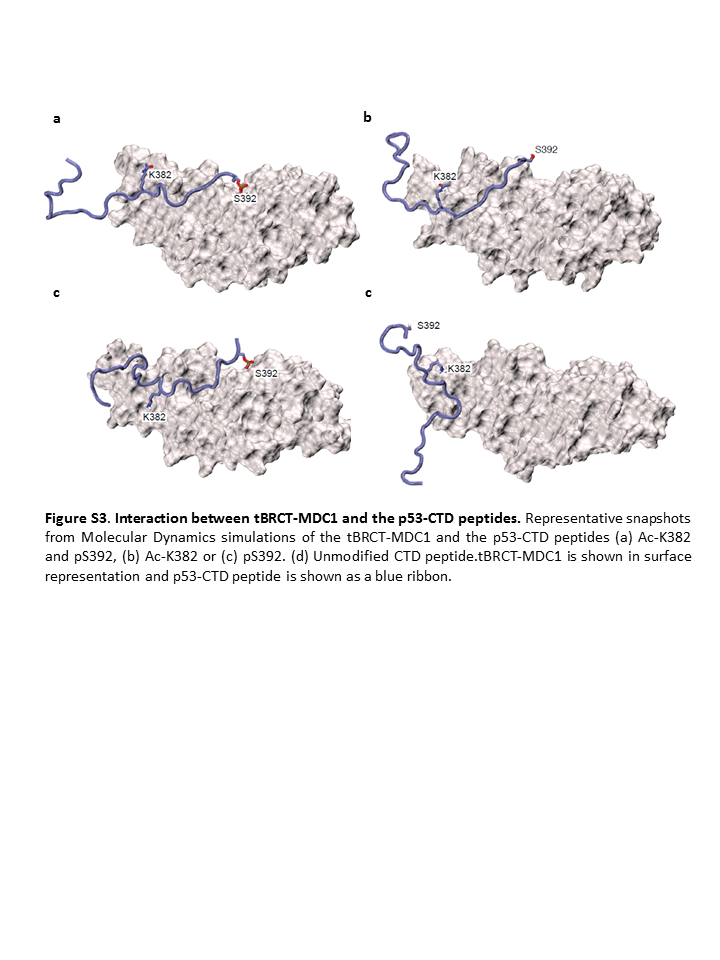

Supplement: Figure S3 — Interaction between tBRCT-MDC1 and the p53-CTD peptides. Representative snapshots from Molecular Dynamics simulations of the tBRCT-MDC1 and the p53-CTD peptides (a) Ac-K382 and pS392, (b) Ac-K382 or (c) pS392. (d) Unmodified CTD peptide.tBRCT-MDC1 is shown in surface representation and p53-CTD peptide is shown as a blue ribbon. (TIF) [file pone.0078472.s003.tif]
